# Surveillance for Silicosis Deaths Among Persons Aged 15–44 Years — United States, 1999–2015

**DOI:** 10.15585/mmwr.mm6628a2

**Published:** 2017-07-21

**Authors:** Jacek M. Mazurek, John M. Wood, Patricia L. Schleiff, David N. Weissman

**Affiliations:** 1 Respiratory Health Division, National Institute for Occupational Safety and Health, CDC.

Silicosis is usually a disease of long latency affecting mostly older workers; therefore, silicosis deaths in young adults (aged 15–44 years) suggests acute or accelerated disease.* To understand the circumstances surrounding silicosis deaths among young persons, CDC analyzed the underlying and contributing causes^†^ of death using multiple cause-of-death data (1999–2015) and industry and occupation information abstracted from death certificates (1999–2013). During 1999–2015, among 55 pneumoconiosis deaths of young adults with *International Classification of Diseases, Tenth Revision* (ICD-10) code J62 (pneumoconiosis due to dust containing silica),^§^ 38 (69%) had code J62.8 (pneumoconiosis due to other dust containing silica), and 17 (31%) had code J62.0 (pneumoconiosis due to talc dust) listed on their death certificate. Decedents whose cause of death code was J62.8 most frequently worked in the manufacturing and construction industries and production occupations where silica exposure is known to occur. Among the 17 decedents who had death certificates listing code J62.0 as cause of death, 13 had certificates with an underlying or a contributing cause of death code listed that indicated multiple drug use or drug overdose. In addition, 13 of the 17 death certificates listing code J62.0 as cause of death had information on decedent’s industry and occupation; among the 13 decedents, none worked in talc exposure–associated jobs, suggesting that their talc exposure was nonoccupational. Examining detailed information on causes of death (including external causes) and industry and occupation of decedents is essential for identifying silicosis deaths associated with occupational exposures and reducing misclassification of silicosis mortality.

Various occupationally associated pulmonary diseases are linked to exposure to silica and silicates, a large class of minerals that includes talc (hydrous magnesium silicate) and other nonfibrous silicate minerals (*1*). Silicosis is caused by inhaling respirable crystalline silica. Occupational exposure to airborne respirable silica particles has been associated with work in mining, quarrying, tunneling, construction, sandblasting, masonry, foundry operations, glass manufacture, ceramic and pottery production, and cement and concrete production and with work with certain materials in dental laboratories (*2*). Newly emerging occupations and tasks, including fabricating and installing quartz-containing engineered stone products and extracting natural gas by hydraulic fracturing also place workers at risk for silicosis.^¶^ Approximately 2.3 million workers might be exposed to respirable crystalline silica in the United States.**

Exposure to talc causes talcosis (talco-silicosis or talco-asbestosis if talc is contaminated with silica or asbestos fibers, respectively); inhalation of talc usually results from occupational exposures during talc mining and milling and during production of ceramics, pharmaceuticals, paint, paper, cosmetics, plastics, roofing, rubber, insecticides, and other products (*3*). Although only 240 workers were employed in talc mining in the United States during 2015 (the number of workers exposed to talc in milling and secondary industries is unknown), 803,000 metric tons of talc were used in various products that year.^††^ Nonoccupational exposure to talc dust has been associated with use of cosmetic talcum powder (*4*) and, importantly, with illicit intravenous or inhalation administration of talc-containing legal or illegal drugs, including marijuana, methamphetamine, methadone, promethazine, cocaine, diazepam, acetaminophen, meperidine, pentazocine, oxymorphone, and heroin (*3*,*5*–*7*).

To investigate silicosis deaths among young adults, ICD-10 codes for underlying and contributing causes of death from the 1999–2015 National Center for Health Statistics’ multiple cause-of-death mortality data were analyzed to provide detailed information on the circumstances surrounding pneumoconiosis deaths among young adults caused by dust containing silica. Time trends were assessed using a linear regression model. Twenty-one states provided copies of actual death certificates^§§^ from 1999 through 2013; usual industry and occupation entries were abstracted from these certificates and were coded using the National Institute for Occupation Safety and Health’s Industry and Occupation Computerized Coding System.^¶¶^

During 1999–2015, a total of 55 young adult decedents had ICD-10 code J62 assigned as either the underlying or a contributing cause of death, including 38 (69%) with ICD-10 subcategory J62.8 listed as the underlying (27) or a contributing (11) cause of death. The mean age of these 38 decedents was 38.6 years; most were males (95%), white (82%), non-Hispanic (74%), and born in the United States (71%) (Table 1). None of these 38 deaths involved multiple drug use or drug overdose; three (8%) had received subcutaneous silicone injections.***

**TABLE 1:** Pneumoconiosis deaths due to dust containing silica (ICD-10 category J62),* among persons aged 15–44 years (n = 55), by patient characteristics, year of death, and ICD-10 subcategory (J62.0 or J62.8^†^) — United States, 1999–2015

Characteristic	J62.0		J62.8	
	Underlying or contributing cause	Underlying cause	Underlying or contributing cause	Underlying cause
**Total**	**17**	**11**	**38**	**27**
**Sex**				
Male	9	7	36	26
Female	8	4	2	1
**Race**				
White	13	9	31	22
Black	3	2	6	4
Other	1	0	1	1
**Ethnicity**				
Hispanic	2	1	10	8
Non-Hispanic	15	10	28	19
**Education**				
≤8 grade	0	0	5	4
9–12	1	1	6	3
High school diploma	8	5	10	9
Some college	1	0	2	2
College degree	2	1	1	0
Unknown	5	4	14	9
**Marital status**				
Married	6	5	18	13
Single/Divorced	11	6	19	13
Unknown	0	0	1	1
**Place of birth**				
United States	17	11	27	18
Outside United States	0	0	11	9
**Year of death**				
1999	1	1	2	1
2000	0	0	5	5
2001	0	0	1	1
2002	1	1	4	3
2003	3	2	3	3
2004	0	0	3	0
2005	0	0	2	1
2006	2	1	4	2
2007	0	0	1	1
2008	0	0	2	2
2009	0	0	1	1
2010	0	0	1	0
2011	1	1	3	2
2012	0	0	0	0
2013	5	3	3	3
2014	1	0	1	0
2015	3	2	2	2
p-value^§^	0.21	0.41	0.09	0.23

**Abbreviation:** ICD-10 =* International Classification of Diseases, Tenth Revision.*

* Decedents with the ICD-10 code J62, pneumoconiosis due to dust containing silica category assigned to their underlying or contributing causes of death.

^†^ ICD-10 code J62 is further divided into subcategories: J62.0 = pneumoconiosis due to talc dust; J62.8 = pneumoconiosis due to other dust containing silica.

^§^ For 1999–2015 time trend (time trends examined using a first-order autoregressive linear regression model).

Seventeen (31%) of the 55 decedents had subcategory J62.0 listed as the underlying (11) or contributing (6) cause of death. The mean age of these decedents was 37.5 years; slightly more than half (9) were male, 13 were white, 15 were non-Hispanic, and all were born in the United States. Thirteen of these 17 deaths involved multiple drug use and drug overdose.^ †††^ The number of pneumoconiosis deaths due to other dust containing silica and due to talc dust among young adults remained stable during 1999–2015 (Table 1).

To evaluate industry and occupation of decedents with a diagnosis of silicosis, CDC obtained death certificates for 47 young adult decedents reported during 1999–2013 from 21 states^§§§^ who had ICD-10 code J62 assigned as the underlying or contributing cause of death. Industry and occupation entries recorded on death certificates were reviewed, including 34 (97%) certificates for 35 deaths with any mention of pneumoconiosis due to other dust containing silica and all certificates for 13 deaths with any mention of pneumoconiosis due to talc dust during 1999–2013. Among the 35 decedents with a diagnosis of pneumoconiosis due to other dust containing silica, the majority were associated with working in the manufacturing (e.g., cut stone and stone product manufacturing industry) (12 [34%]) and construction (7 [20%]) industry sectors; 11 (31%) were working in production (e.g., crushing, grinding, polishing, mixing, and blending workers) occupations; five (14%) in construction and extraction occupations; and three (9%) as brickmasons and blockmasons (Table 2). These industries and occupations have well-established associations with exposure to crystalline silica (*2*). Among the 13 decedents whose death certificates included any mention of pneumoconiosis due to dust containing talc, none was employed in an industry or occupation traditionally associated with exposure to talc. Ten of these 13 decedents were assigned codes indicating multiple drug use or drug overdose. Among these 10 decedents, three worked in the health care and social assistance industry (offices of dentists, ambulatory health care services, and general medical and surgical hospitals) (Table 3).

**TABLE 2:** Deaths due to other dust containing silica (ICD-10 subcategory J62.8),* among persons aged 15–44 years (n = 35), by year of death, age, industry and occupation,^†^ and assignment of code J62.8 as the underlying cause — United States, 1999–2013

Year of death	Age (yrs)	Industry	Occupation	J62.8 code listed as the underlying cause
1999	40	Cut stone and stone product manufacturing	Crushing, grinding, and polishing machine setters, operators, and tenders	Yes
1999	43	Commercial and institutional building construction	Cement masons and concrete finishers	No
2000	32	Cut stone and stone product manufacturing	Etchers and engravers	Yes
2000	34	Unknown, blank, inadequate information	Sandblaster^§^	Yes
2000	41	Manufacturing	Production workers, all other	Yes
2000	41	Nonmetallic mineral mining and quarrying	Loading machine operators, underground mining	Yes
2000	43	Services to buildings and dwellings	Janitors and cleaners, except maids and housekeeping cleaners	Yes
2001	39	Construction of buildings	Brickmasons and blockmasons	Yes
2002	40	All other miscellaneous chemical product and preparation manufacturing	Industrial production managers	No
2002	41	Masonry contractors	Brickmasons and blockmasons	Yes
2002	43	Vitreous china, fine earthenware, and other pottery product manufacturing	Production workers, all other/Machine feeders	Yes
2002	44	Unknown, blank, inadequate information	Unknown, blank, inadequate information	Yes
2003	22	Construction	Construction laborers	Yes
2003	31	Retail trade	First-line supervisors of retail sales workers	Yes
2003	35	Unknown, blank, inadequate information	Unknown, blank, inadequate information	Yes
2004	41	Cut stone and stone product manufacturing	Laborers and freight, stock, and material movers, hand	No
2004	42	Nonmetallic mineral product manufacturing	Production workers, all other	No
2004	44	Ferrous metal foundries	Crushing, grinding, and polishing machine setters, operators, and tenders	No
2005	36	Tile and terrazzo contractors	Brickmasons and blockmasons	Yes
2005	41	Cement and concrete product manufacturing	Production workers, all other	No
2006	38	Unknown, blank, inadequate information	Unknown, blank, inadequate information	Yes
2006	41	Cut stone and stone product manufacturing	Crushing, grinding, and polishing machine setters, operators, and tenders	Yes
2006	42	Agencies, brokerages, and other insurance related activities	Social workers, all other	No
2006	43	Unknown, blank, inadequate information	Unknown, blank, inadequate information	No
2007	34	Electric power generation, transmission and distribution	Painting, coating, and decorating workers	Yes
2008	43	Cut stone and stone product manufacturing	Etchers and engravers	Yes
2008^¶^	34	Unknown, blank, inadequate information	Unknown, blank, inadequate information	Yes
2009	32	Janitorial services	Janitors and cleaners, except maids and housekeeping cleaners	Yes
2010	37	Nonpaid workers	Did not work	No
2011	34	General freight trucking	Heavy and tractor-trailer truck drivers	No
2011	35	Manufacturing	Production workers, all other	Yes
2011	44	Unknown, blank, inadequate information	Unknown, blank, inadequate information	Yes
2013	36	Construction	Industrial production managers	Yes
2013	41	Construction of buildings	Operating engineers and other construction equipment operators	Yes
2013	44	Other miscellaneous durable goods merchant wholesalers	Industrial truck and tractor operators	Yes

**Abbreviation:** ICD-10* = International Classification of Diseases, Tenth Revision.*

* Assigned as either the underlying or contributing cause of death in the National Center for Health Statistics (NCHS) multiple cause-of-death data.

^†^ Usual industry and occupation entries on death certificates for 34 (97%) of 35 pneumoconiosis deaths caused by other dust containing silica reported for 1999–2013 were available for review and coded using North American Industry Classification System and 2010 Standard Occupational Classification codes (https://wwwn.cdc.gov/niosh-nioccs/default.aspx).

^§^ Because industry was not known, this occupation could be coded as 1) cleaners of vehicles and equipment, 2) crushing, grinding, and polishing machine setters, or 3) operators, and tenders construction laborers.

^¶^ Death certificate was unavailable for review; information on year of death, age, and codes assigned as the underlying cause of death from the NCHS multiple cause-of-death data.

**TABLE 3:** Deaths due to talc dust (ICD-10 subcategory J62.0)* among persons aged 15–44 years (n = 13), by year of death, age, industry and occupation,^†^ and assignment of code indicating multiple drug use or drug overdose^§^ — United States, 1999–2013

Year of death	Age (yrs)	Industry	Occupation	Multiple drug use or drug overdose codes listed
1999	37	Nonpaid workers	Homemakers	Yes
2002	41	Voluntary health organizations	Secretaries and administrative assistants, except legal, medical, and executive	No
2003	37	Glass and glass product manufacturing	Glaziers	Yes
2003	40	Offices of dentists	Dental laboratory technicians	Yes
2003	41	Ambulatory health care services	Emergency medical technicians and paramedics	Yes
2006	19	Nonpaid workers	Students	No
2006	40	Nonpaid workers	Did not work	Yes
2011	41	Construction	Operating engineers and other construction equipment operators	Yes
2013	34	Computer systems design and related services	Computer occupations, all other	No
2013	36	Unknown, blank, inadequate information	Driver/sales workers	Yes
2013	36	All other specialty trade contractors	Construction managers	Yes
2013	43	Nonpaid workers	Did not work	Yes
2013	44	General medical and surgical hospitals	Registered nurses	Yes

**Abbreviation:** ICD-10 =* International Classification of Diseases, Tenth Revision.*

* Assigned as either the underlying or contributing cause of death in the National Center for Health Statistics (NCHS) multiple cause-of-death data.

^†^ Usual industry and occupation entries on death certificates for all 13 (100%) pneumoconiosis deaths due to talc dust reported for 1999–2013 were available for review and coded using North American Industry Classification System and 2010 Standard Occupational Classification codes (https://wwwn.cdc.gov/niosh-nioccs/default.aspx).

^§^ ICD-10 codes indicating multiple drug use of drug overdose: X42 (accidental poisoning by and exposure to narcotics and psychodysleptics [hallucinogens], not elsewhere classified); X44 (accidental poisoning by and exposure to other and unspecified drugs, medicaments and biologic substances); F19 (multiple drug use and use of other psychoactive substances [F19.1 (harmful use)], [F19.9 (unspecified mental and behavioral disorder)]); T39 (poisoning by nonopioid analgesics, antipyretics and antirheumatics, including T39.8 [other nonopioid analgesics and antipyretics, not elsewhere classified]); T40 (poisoning by narcotics and psychodysleptics [hallucinogens] [T40.2 (other opioids), T40.3 (methadone), T40.6 (other and unspecified narcotics)]); T42 (poisoning by antiepileptic, sedative-hypnotic and antiparkinsonism drugs, including T42.4 [benzodiazepines]) and T42.6 [other antiepileptic and sedative-hypnotic drugs]); X40 (accidental poisoning by and exposure to nonopioid analgesics, antipyretics, and antirheumatics) listed as the underlying or a contributing cause in the NCHS multiple cause-of-death data.

## Discussion

Among 55 deaths in young adults reported for 1999–2015 with ICD-10 code J62 assigned as either the underlying or a contributing cause of death, 13 were coded as subcategory J62.0, indicating exposure to talc dust, and in most of these cases, the underlying or contributing cause-of-death codes also indicated multiple drug use or drug overdose. These deaths likely represent nonoccupational pulmonary talcosis caused by illicit inhalation or intravenous administration of talc-contaminated drugs (*3*,*5*–*7*). Eight of the 13 pneumoconiosis deaths attributed to talc dust were associated with multiple drug use and drug overdose occurred during 2010–2015, and coincided with the expanding epidemic of drug overdose deaths in the United States (*8*).

The remaining two thirds of silicosis deaths were coded as J62.8. Among silicosis deaths reported for 1999–2013, manufacturing or construction industries, both of which are known to be associated with exposures to silica-containing dust, were frequently listed on death certificates for these decedents. Three decedents had a history of subcutaneous silicone injections and likely were erroneously assigned code J62.8 as the underlying cause of death.

The findings in this report are subject to at least five limitations. First, no information on silica exposure intensity or duration is listed on death certificates. Silicosis-associated deaths in young adults should be considered sentinel cases, potentially resulting from high exposures that cause short latency to disease onset and rapid disease progression. Second, lifetime occupational histories of decedents were not collected, and the usual industry and occupation listed on death certificates might not accurately represent the industry or occupation where the hazardous silica exposure occurred. However, there is a generally good agreement of industry and occupation information on death certificates compared with that from other sources (*9*). Third, industry and occupation information was only available for 40 (83%) and 42 (88%) decedents, respectively, who were included in reports during 1999–2013. Fourth, pneumoconiosis as a cause of death might have been misclassified or under- or overreported. Finally, increased recognition of drug-related deaths, improvements in testing, and reporting of deaths involving drug use might have contributed to the high frequency of reported multiple drug use and drug overdose among pneumoconiosis deaths due to talc. The continuing occurrence of pneumoconiosis deaths due to other dust containing silica indicates the need for maintaining measures to limit workplace exposure to respirable crystalline silica. Primary prevention of pneumoconioses relies on elimination or effective control of exposures (https://www.cdc.gov/niosh/topics/hierarchy/). Effective silicosis prevention strategies for employers are available from the Occupational Safety and Health Administration (https://www.osha.gov/silica/) and CDC (https://www.cdc.gov/niosh/topics/silica). The occurrence of pneumoconiosis deaths due to talc associated with multiple drug use and drug overdose reinforces the need for a multifaceted, collaborative clinical, public health, public safety, and law enforcement approach to the drug overdose epidemic (*8*). Examining detailed information on causes of death, including external causes, along with industry and occupation of decedents, is essential for identifying silicosis deaths associated with occupational exposures and reducing misclassification of silicosis mortality.

**Summary**

**What is already known about this topic?**

Various preventable occupational pulmonary diseases are associated with exposure to respirable particles of crystalline silica and other silicate materials, one of which is talc (hydrous magnesium silicate). Detailed information on the circumstances surrounding deaths of silicosis decedents is needed to better target intervention and prevention measures.

**What is added by this report?**

During 1999–2015, among 55 decedents aged 15–44 years who had pneumoconiosis due to dust containing silica assigned as either the underlying or contributing cause of death, 38 (69%) were assigned pneumoconiosis due to other dust containing silica, and 17 (31%) were assigned pneumoconiosis due to talc dust. Decedents with pneumoconiosis due to other dust containing silica had manufacturing or construction industry frequently listed as the occupation on their death certificates; both industries are well known to be associated with exposures to silica-containing dust. Among 17 decedents with pneumoconiosis due to talc dust, 13 (76%) involved multiple drug use or drug overdose and none worked in talc exposure-associated jobs.

**What are the implications for public health practice?**

Among deaths in persons aged 15–44 years attributed to pneumoconiosis due to dust containing silica, nearly one third had pneumoconiosis due to talc dust. Most of these cases likely represent nonoccupational exposure to talc. Examining detailed information on causes of death, including external causes, along with industry and occupation of decedents is essential for identifying silicosis deaths associated with occupational exposures and reducing misclassification of silicosis mortality.

## Footnotes

* Chronic silicosis, the most common form of silicosis, occurs after exposure to relatively low silica concentrations for >10 years. Accelerated silicosis occurs after 5–10 years of exposure to higher silica levels, and acute silicosis can occur after only weeks or months of exposure to extremely high silica concentrations.

† Records included on the entity axis that reflect the placement of each condition on the certificate for each decedent (https://www.cdc.gov/nchs/data/dvs/2b_2016.pdf); each record includes codes for one underlying cause of death (the disease or injury that initiated the chain of events that led directly and inevitably to death) and up to 20 contributing causes of death. https://webappa.cdc.gov/ords/norms-glossary.html.

^ §^ *International Classification of Diseases, Tenth Revision* (ICD-10) code J62, pneumoconiosis due to dust containing silica category, is further subdivided into pneumoconiosis due to talc dust (J62.0) and pneumoconiosis due to other dust containing silica (J62.8). http://apps.who.int/classifications/icd10/browse/2016/en#/J62.

¶ https://www.cdc.gov/niosh/topics/silica.

** https://www.osha.gov/silica/.

†† https://minerals.usgs.gov/minerals/pubs/mcs/2017/mcs2017.pdf.

§§ CDC requested death certificates from 22 states for 48 young (aged 15–44 years) decedents reported for 1999–2013 who had ICD-10 code J62 (pneumoconiosis due to dust containing silica) assigned as the underlying or contributing cause of death. Among these, 47 (received from 21 states) were available for review. Seven additional deaths with code J62 assigned to cause of death among young adults were identified in the National Center for Health Statistics multiple cause-of-death data for 2014 and 2015 (when the new data became available).

¶¶ Industry and occupation entries were coded using North American Industry Classification System and 2010 Standard Occupational Classification codes. https://www.cdc.gov/niosh/topics/coding/overview.html.

*** Three (8%) decedents had the underlying cause of death coded as pneumoconiosis due to other dust containing silica (J62.8) and had one or more contributing causes of death coded as the following: T65.8 (toxic effect of other and unspecified substances); T80.9 (complications following infusion, transfusion, and therapeutic injection); Y56.3 (topical agents primarily affecting skin and mucous membrane and ophthalmological, otorhinolaryngological, and dental drugs); X49 (accidental poisoning by and exposure to other and unspecified chemicals and noxious substances). All three decedents received subcutaneous silicone injections.

††† Five (29%) decedents had the underlying cause of death coded as X42 (accidental poisoning by and exposure to narcotics and psychodysleptics [hallucinogens], not elsewhere classified; n = 1) or X44 (accidental poisoning by and exposure to other and unspecified drugs, medicaments and biological substances; n = 4). Eight (47%) decedents had one or more contributing causes of death coded as F19 (multiple drug use and use of other psychoactive substances, including F19.1 [harmful use] or F19.9 [unspecified mental and behavioral disorder]); T39 (poisoning by nonopioid analgesics, antipyretics and antirheumatics, including T39.8 [other nonopioid analgesics and antipyretics, not elsewhere classified]); T40 (poisoning by narcotics and psychodysleptics [hallucinogens], including T40.2 [other opioids], T40.3 [methadone], T40.6 [other and unspecified narcotics]); T42 (poisoning by antiepileptic, sedative-hypnotic and antiparkinsonism drugs), including T42.4 [benzodiazepines], T42.6 [other antiepileptic and sedative-hypnotic drugs]); or X40 (accidental poisoning by and exposure to nonopioid analgesics, antipyretics, and antirheumatics).

§§§ Alabama, Arizona, Arkansas, California, Georgia, Illinois, Indiana, Kansas, Kentucky, Louisiana, Michigan, Minnesota, Mississippi, New Jersey, New Mexico, North Carolina, Ohio, Pennsylvania, Tennessee, Texas, and Washington.
